# Triglyceride to HDL-Cholesterol Ratio and the Incident Risk of Ischemic Heart Disease Among Koreans Without Diabetes: A Longitudinal Study Using National Health Insurance Data

**DOI:** 10.3389/fcvm.2021.716698

**Published:** 2021-08-17

**Authors:** Byoungjin Park, Dong Hyuk Jung, Hye Sun Lee, Yong Jae Lee

**Affiliations:** ^1^Department of Family Medicine, Yongin Severance Hospital, Seoul, South Korea; ^2^Department of Family Medicine, Yonsei University College of Medicine, Seoul, South Korea; ^3^Biostatistics Collaboration Unit, Department of Research Affairs, Yonsei University College of Medicine, Seoul, South Korea; ^4^Department of Family Medicine, Gangnam Severance Hospital, Seoul, South Korea

**Keywords:** triglyceride to HDL-cholesterol ratio, cardiometabolic risk, insulin resistance, ischemic heart disease, cohort study

## Abstract

**Background:** Early insulin resistance without diabetes can cause cardiovascular disease, which is a public health challenge. This study aimed to investigate the effects of the triglyceride to high-density lipid (HDL)-cholesterol ratio (TG/HDL-C), which could reflect insulin resistance from the beginning, on the incident risk of ischemic heart disease (IHD).

**Methods:** We assessed 16,455 individuals (8,426 men and 8,029 women) without diabetes in a community-dwelling Korean cohort using National Health Insurance data. Participants were classified based on the TG/HDL-C quartiles. Using multivariate Cox proportional-hazards regression models, we prospectively examined the hazard ratios (HRs) with 95% confidence intervals (CIs) for IHD over 50 months after baseline enrolment.

**Results:** During the follow-up period, 321 (2.0%) participants developed IHD. After adjusting for potential confounding variables, the HRs of IHD for TG/HDL-C quartiles were 1.00, 1.61 (95% CI, 1.05–2.48), 1.85 (95% CI, 1.21–2.81), and 2.29 (95% CI, 1.50–3.51), respectively. Compared with men, women showed higher HRs for the risk of incident IHD in the fourth quartile [HR (95% CI) = 2.98 [1.50–5.88] and 1.80 [1.02–3.17], respectively). Compared with metabolic syndrome, TG/HDL-C had a more powerful predictive value for IHD.

**Conclusions:** In Koreans without diabetes, an increased TG/HDL-C precedes future IHD. Additionally, sex differences may merit serious consideration when interpreting TG/HDL-C for assessing cardiovascular risks in clinical practice.

## Introduction

Ischemic heart disease (IHD) is a significant challenge among middle-aged individuals without diabetes. From an epidemiological perspective, patients without diabetes who have IHD can have a poorer prognosis than patients with diabetes (1–3). There may be a significant decline in insulin sensitivity over approximately 5 years as a natural course of β-cell function, even among individuals with normal glucose tolerance or in a pre-diabetic state (4). Additionally, coronary heart disease can begin in early-stage insulin resistance (5), which may be overlooked in clinical settings. Therefore, management of IHD risk factors in the preclinical stage is important from a public health perspective, which facilitates disease prevention and delays IHD progression.

Individuals with early insulin resistance often have increased and decreased triglyceride (TG) and high-density lipoprotein cholesterol (HDL-C) levels, respectively (6). Epidemiological studies have reported the predictive utility of the TG to HDL-C ratio (TG/HDL-C) for type 2 diabetes, which suggests that it is a simple and more accessible marker of insulin resistance from onset compared with homeostasis model assessment of insulin resistance or hyperinsulinemic-euglycemic clamp testing (7–9). Furthermore, TG/HDL-C is closely associated with small dense low-density lipoprotein cholesterol (LDL-C) levels, which have atherogenic properties (10, 11).

A recent study reported that a higher TG/HDL-C ratio is associated with the subclinical burden of coronary atherosclerosis (12). Previous prospective studies have primarily focused on cardiovascular mortality or individuals with some cardiovascular risks (13–16). Additionally, sex differences in these relationships remain unclear. This prospective study aimed to investigate the relationship between the TG/HDL-C ratio and incident IHD in a large-scale, community-dwelling, Korean cohort without diabetes, using National Health Insurance Service data.

## Materials and Methods

### Study Design and Participants

**Figure 1:**
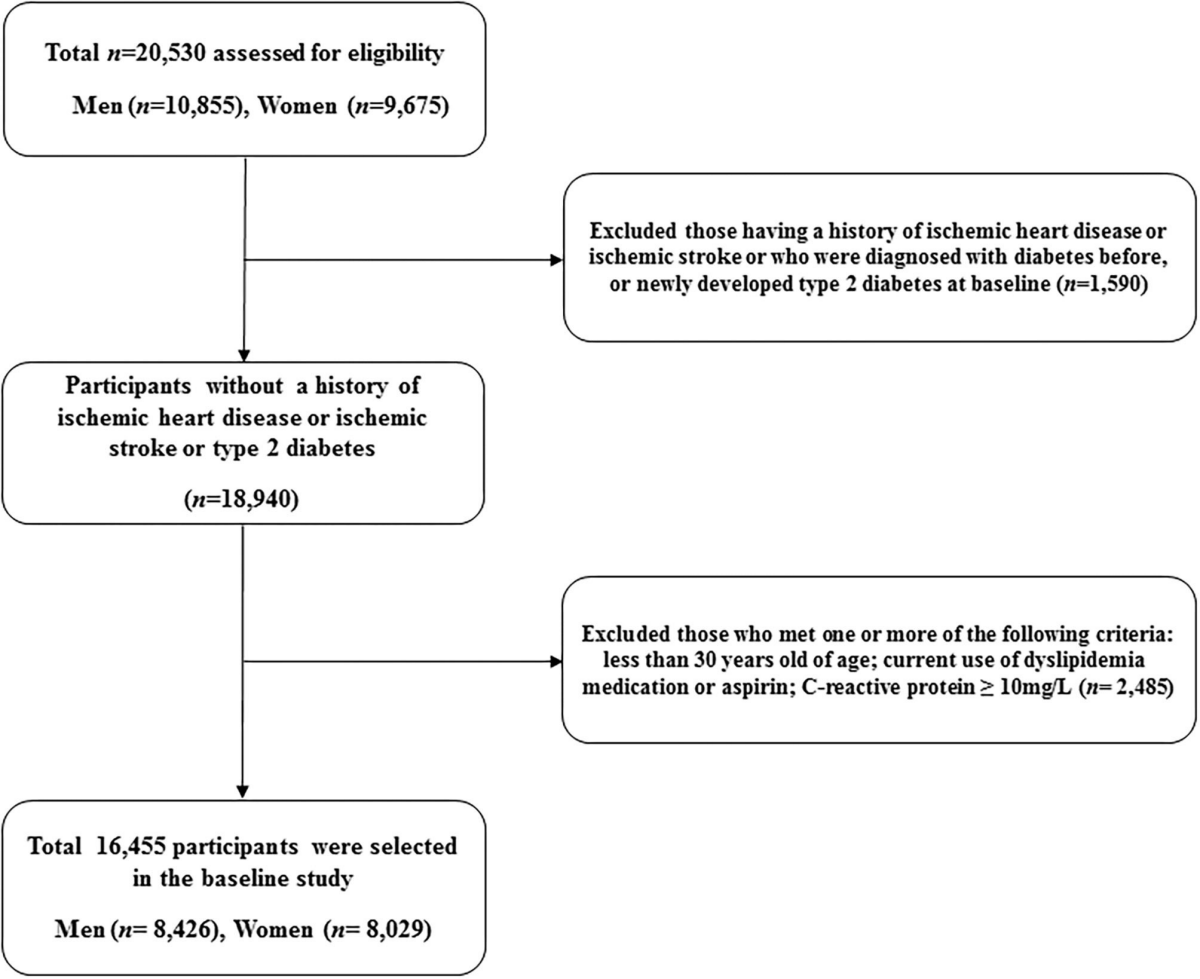
Flowchart for the selection of study participants.

This study is a derivative of the Health Risk Assessment Study (HERAS), which was designed to identify and explore surrogate indicators for cardiometabolic diseases among Korean adults (17, 18). The HERAS cohort is comprised of 20,530 participants who sequentially visited the Health Promotion Center, Yonsei University Gangnam Severance Hospital, for health examination between November 1, 2006, and June 8, 2010. Among the participants identified at baseline, we excluded 1,590 individuals previously diagnosed with angina pectoris, myocardial infarction, ischemic stroke, diabetes mellitus, or newly developed type 2 diabetes (defined as a fasting plasma glucose level ≥ 126 mg/dl). Additionally, we excluded participants according to the following criteria: aged < 30 years, current use of dyslipidemia medication or aspirin, and high-sensitivity C-reactive protein (hsCRP) level ≥ 10 mg/L (*n* = 2,485). Consequently, 16,455 individuals were included in the final analysis (Figure 1).

### Data Collection

Each participant completed a lifestyle behavior and medical history questionnaire, which included information regarding cigarette smoking, alcohol intake, and physical activity. Smoking status was defined using the following categories: non-smoker, ex-smoker, and current smoker. Regular alcohol intake was defined as an alcohol consumption rate of ≥ 140 g/week. Regular exercise was defined as moderate physical activity ≥ thrice per week. Bodyweight and height were measured to the nearest 0.1 kg and 0.1 cm, respectively, in light indoor clothing without footwear. The body mass index was calculated as the weight in kilograms divided by the square of the height in meters (kg/m^2^). Systolic and diastolic blood pressures were measured on the patient's right arm using a standard mercury sphygmomanometer in the sitting position after 10 min of rest (Baumanometer, W.A. Baum Co., Inc., Copiague, NY, USA). The mean arterial pressure was calculated from the systolic and diastolic blood pressures and weighted as 1/3 systolic and 2/3 diastolic blood pressure (19). Blood samples were obtained from the antecubital vein after overnight 12-h fasting. Fasting plasma glucose, total cholesterol, triglyceride, and HDL-C levels were measured through enzymatic methods using a Hitachi 7,600 automated chemistry analyzer (Hitachi Co., Tokyo, Japan). Serum hsCRP levels were measured with a Roche/Hitachi 912 System using a latex-enhanced immunoturbidimetric method (Roche Diagnostics, Indianapolis, IN, USA). Hypertension was defined as a systolic and diastolic blood pressure ≥ 140 and ≥ 90 mmHg, respectively, or current hypertension medication use (20). Impaired fasting glucose was defined as a fasting plasma glucose level of 100–126 mg/dl (21). Metabolic syndrome was defined using the modified National Cholesterol Education Program Adult Treatment Panel III. Since we did not measure the waist circumference, obesity was defined as BMI ≥ 25 kg/m^2^, as suggested by the position statement of the American College of Endocrinology (22). Metabolic syndrome was defined as the presence of ≥ 3 of the following risk factors: obesity with BMI ≥ 25.0 kg/m^2^, elevated systolic blood pressure ≥ 130 mmHg, elevated diastolic blood pressure ≥85 mmHg, or using an antihypertensive medication; high fasting plasma glucose levels ≥ 100 mg/dl or using diabetes medication; triglyceride levels ≥ 150 mg/dl; and HDL cholesterol levels < 40 mg/dl and <50 mg/dl for men and women, respectively. TG/HDL-C was calculated as TG (mg/dl) divided by HDL-C level (mg/dl). TG/HDL-C values were categorized into quartiles as follows: Q1 ( ≤ 1.25), Q2 (1.26–1.98), Q3 (1.99–3.24), and Q4 (≥ 3.25).

### Study Outcomes

The primary outcome was IHD, which comprised of angina pectoris (ICD-10 code I20) or acute myocardial infarction (ICD-10 code I21) occurring after study enrolment (17, 18). To define baseline and post-survey outcomes, we linked a personal 13-digit identification number for each participant that was assigned using the Health Insurance Review and Assessment Data (HIRA) in Korea, which is a repository of claims data collected while reimbursing healthcare providers, from November 1, 2006, to December 31, 2010.

### Statistical Analysis

Baseline characteristics were compared among the TG/HDL-C quartiles using analysis of variance (ANOVA) and the chi-squared test for continuous and categorical variables, respectively. Moreover, we performed sex-based comparisons of the clinical and biochemical characteristics using independent two-sample *t*-tests and a chi-square test. Kaplan–Meier curves were used to assess the cumulative incidence of IHD. The log-rank test was used for among-group comparisons of the distribution of the cumulative IHD incidence. In multivariate analysis, after setting the lowest TG/HDL-C quartile as a reference group, the hazard ratios (HRs) and 95% confidence intervals (CIs) for incident IHD were calculated using the Cox proportional hazards regression model after adjusting for potential confounding variables. Pairwise comparisons of receiver-operating characteristic (ROC) curves were used to compare the areas under ROC curves (AUC) for IHD incidence based on TG/HDL-C, presence of metabolic syndrome, and the number of metabolic syndrome components. Furthermore, we used AUC values to test the sensitivity and specificity of the biomarkers for predicting IHD. All statistical analyses were performed using SAS version 9.4 software (; SAS Institute Inc., Cary, NC, USA) and all statistical tests were two-sided. Statistical significance was set at *p* < 0.05.

## Results

### Baseline Characteristics of the Study Population

Table 1 shows the baseline characteristics of the study population (*n* = 16,455; 8,426 men and 8,029 women) based on the TG/HDL-C quartiles. The mean age, BMI, TG levels, HDL-C levels, and TG/HDL-C were 46.1 ± 9.5 years, 23.4 ± 3.0 kg/m^2^, 124.2 ± 84.9, 53.2 ± 12.6, and 2.64 ± 2.42 mg/dl, respectively.

**Table 1:** Baseline characteristics of the study population according to the TG/HDL-C quartiles.

**Characteristics**		**TG/HDL-C quartiles**					
	**Overall**	**Q1**	**Q2**	**Q3**	**Q4**	***P-value*** ^**a**^	***Post-hoc*** ^**b**^
	**(***n =*** 16,455)**	**(***n =*** 4,112)**	**(***n =*** 4,147)**	**(***n =*** 4,081)**	**(***n =*** 4,115)**		
TG/HDL-C	2.64 ± 2.42	≤ 1.25	1.26–1.98	1.99–3.24	≥ 3.25		
Age (years)	46.1 ± 9.5	44.4 ± 9.2	46.3 ± 9.5	47.0 ± 9.5	46.6 ± 9.6	<0.001	a,b,c,d
Male sex (%)	51.2	24.8	43.0	61.2	76.0	<0.001	-
Body mass index (kg/m^2^)	23.4 ± 3.0	21.5 ± 2.5	22.8 ± 2.7	24.1 ± 2.8	25.1 ± 2.7	<0.001	a,b,c,d,e,f
Systolic blood pressure (mmHg)	121.9 ± 15.5	115.8 ± 14.5	120.1 ± 15.1	124.2 ± 15.0	127.7 ± 14.7	<0.001	a,b,c,d,e,f
Diastolic blood pressure (mmHg)	76.2 ± 10.1	72.1 ± 9.4	75.0 ± 9.7	77.7 ± 9.8	80.1 ± 9.6	<0.001	a,b,c,d,e,f
Fasting plasma glucose (mg/dl)	91.4 ± 9.8	87.8 ± 8.8	90.5 ± 8.9	92.7 ± 9.6	94.7 ± 10.3	<0.001	a,b,c,d,e,f
Total cholesterol (mg/dl)	190.3 ± 33.3	181.7 ± 30.7	185.8 ± 31.5	194.0 ± 33.0	199.6 ± 34.9	<0.001	a,b,c,d,e,f
Triglyceride (mg/dl)	124.2 ± 84.9	60.6 ± 13.1	87.9 ± 16.4	123.3 ± 23.8	225.2 ± 110.8	<0.001	a,b,c,d,e,f
HDL-cholesterol (mg/dl)	53.2 ± 12.6	66.0 ± 11.3	55.5 ± 9.2	49.2 ± 8.1	42.1 ± 7.1	<0.001	a,b,c,d,e,f
C-reactive protein (mg/L)	1.0 ± 1.3	0.7 ± 1.1	0.9 ± 1.3	1.2 ± 1.4	1.4 ± 1.5	<0.001	a,b,c,d,e,f
Current smoker (%)	24.7	10.2	18.6	28.8	40.9	<0.001	-
Alcohol drinking (%)	43.3	35.0	40.0	45.9	52.4	<0.001	-
Regular exercise (%)	30.9	33.5	33.8	30.1	26.3	<0.001	-
Hypertension (%)	20.3	10.9	16.1	23.7	30.7	<0.001	-
Impaired fasting glucose (%)	18.0	12.0	19.7	29.3	39.1	<0.001	-
Metabolic syndrome (%)	12.1	0.6	2.4	8.0	37.5	<0.001	-

a *P-values were calculated using 1-way ANOVA or Pearson's chi-square test*.

b *Post-hoc analysis with the Bonferroni method: a, Q1 vs. Q2; b, Q1 vs. Q3; c, Q1 vs. Q4; d, Q2 vs. Q3; e, Q2 vs. Q4; and f, Q3 vs. Q4*.

The group with the highest TG/HDL-C quartile showed the highest mean values of BMI, mean arterial pressure, fasting plasma glucose, total cholesterol, and hsCRP levels, as well as the lowest mean HDL-C levels. Moreover, this group showed the highest proportions of current smokers and alcohol drinkers, as well as the lowest proportion of individuals involved in regular exercise. The prevalence of hypertension, impaired fasting glucose, and metabolic syndrome were 20.3, 18.0, and 12.1%, respectively, which gradually increased according to TG/HDL-C quartiles. There was no sex difference in the age distribution; however, men showed a higher TG/HDL-C than women (3.33 ± 2.91 vs. 1.91 ± 1.44; Table 2).

**Table 2:** Clinical and biochemical characteristics according to sex.

	**Men**	**Women**	***P*** -value^*^
	**(***n =*** 8,426)**	**(***n =*** 8,029)**	
TG/HDL-C	3.33 ± 2.91	1.91 ± 1.44	<0.001
Age (years)	45.9 ± 9.5	46.2 ± 9.5	0.037
Body mass index (kg/m^2^)	24.3 ± 2.8	22.4 ± 2.9	<0.001
Systolic blood pressure (mmHg)	126.3 ± 14.3	117.4 ± 15.4	<0.001
Diastolic blood pressure (mmHg)	79.4 ± 9.4	72.9 ± 9.7	<0.001
Mean arterial pressure (mmHg)	95.0 ± 10.7	87.7 ± 11.2	<0.001
Fasting plasma glucose (mg/dl)	93.5 ± 9.9	89.3 ± 9.2	<0.001
Total cholesterol (mg/dl)	192.4 ± 32.8	188.0 ± 33.7	<0.001
Triglyceride (mg/dl)	147.9 ± 100.0	99.3 ± 55.4	<0.001
HDL-cholesterol (mg/dl)	49.0 ± 11.0	57.7 ± 12.7	<0.001
C-reactive protein (mg/L)	1.2 ± 1.4	0.9 ± 1.2	<0.001
Current smoker (%)	42.2	5.5	<0.001
Alcohol drinking (%)	60.5	49.9	<0.001
Regular exercise (%)	32.0	29.8	0.002
Hypertension (%)	26.1	14.3	<0.001
Impaired fasting glucose (%)	23.6	12.1	<0.001
Metabolic syndrome (%)	13.0	11.1	<0.001

* *p values were calculated using an independent two-sample t-test or Pearson's chi-squared tests*.

### Cumulative Ischemic Heart Disease Incidence

**Figure 2:**
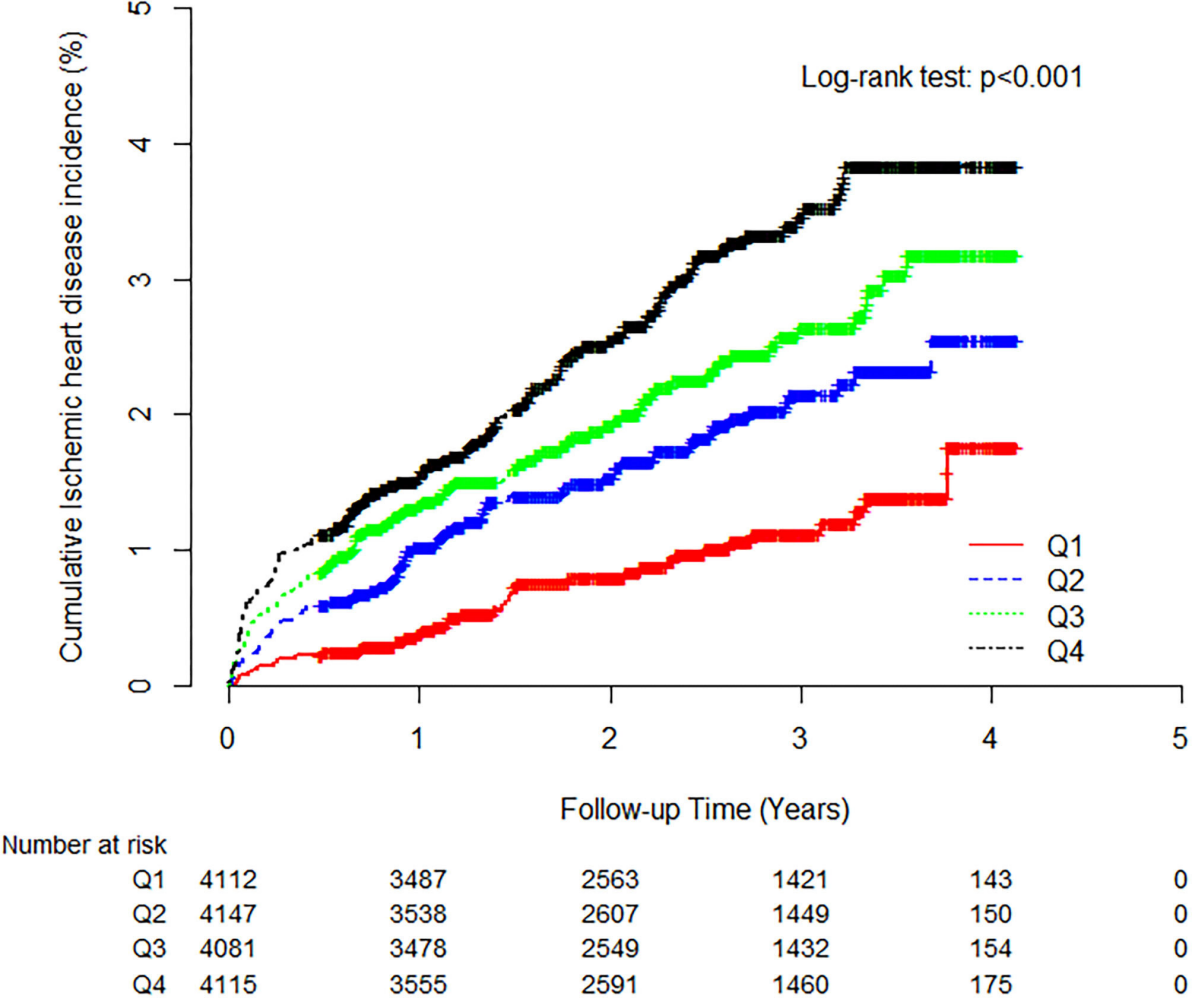
Kaplan-Meier plots indicating the cumulative probability of being diagnosed with ischemic heart disease after the baseline survey.

During the follow-up period, 321 (2.0%, 321/16,455) individuals developed IHD. The IHD incidence rate (per 1,000 person-years) was positively correlated with the TG/HDL-C quartiles. The higher TG/HDL-C groups showed a significantly increased cumulative IHD incidence over 50 months following the baseline survey (log-rank test, *P* < 0.001) (Figure 2).

### Hazard Ratios for Incident Ischemic Heart Diseases

**Figure 3:**
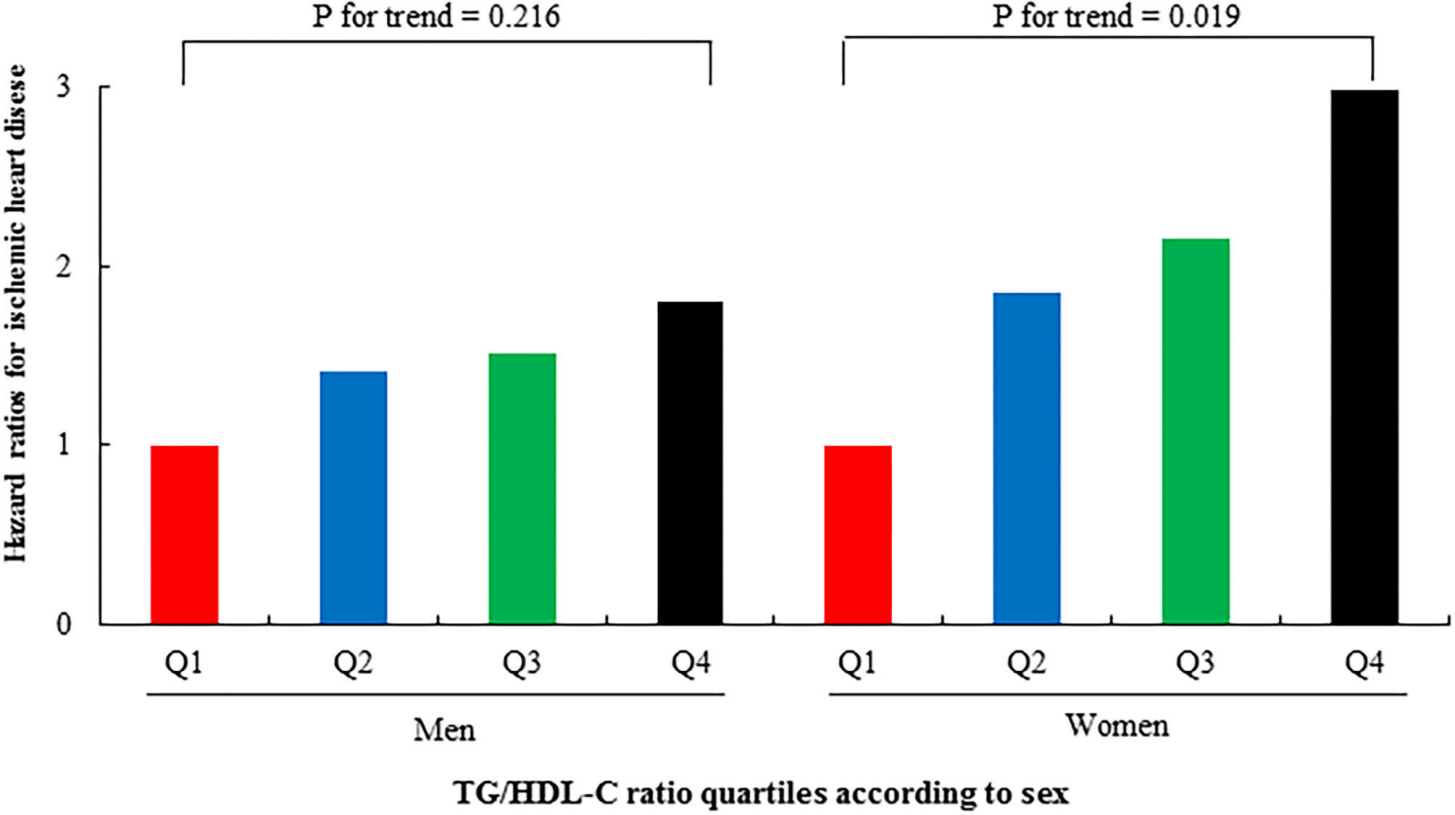
Hazard ratios (95% CIs) for incident IHD according to TG/HDL-C quartiles after adjusting for potential confounding variables according to sex.

Tables 3, 4 show the results of the Cox proportional hazards regression analysis for predicting IHD based on the TG/HDL-C quartiles. Compared with the reference first TG/HDL-C quartile, the HRs of incident IHD for the second, third, and fourth quartiles increased in a dose-dependent manner. The HRs for incident IHD were 1.64 (95% CI, 1.07–2.52), 1.85 (95% CI, 1.21–2.83), and 2.35 (95% CI, 1.53–3.60) in the second, third and fourth TG/HDL-C quartiles, respectively, after adjusting for age, sex, BMI, smoking status, alcohol intake, and physical activity. Similarly, these longitudinal positive associations were observed after additionally adjusting for systolic blood pressure, diastolic blood pressure, fasting plasma glucose, hsCRP levels, and hypertension medication. The corresponding adjusted HR for the highest vs. lowest TG/HDL-C quartile was 2.26 (95% CI, 1.47–3.48). Sex-based subgroup analysis revealed that the HRs of incident IHD for the fourth quartile were higher in women than in men [HR (95% CI) = 2.98 [1.51–5.89] and 1.81 [1.03–3.20], respectively) (Model 3, Figure 3).

**Table 3:** Univariate and multivariate Cox proportional-hazards regression models for incident ischemic heart disease.

**Variables**	**Univariate**			**Multivariate**		
	**HRs**	**95% CIs**	***P*** **-value**	**HRs**	**95% CIs**	***P*** **-value**
Age, years	1.07	1.06–1.08	<0.001	1.06	1.04–1.07	<0.001
Male sex, yes or no	1.60	1.28–2.01	<0.001	1.50	1.06–2.12	0.023
Body mass index, kg/m^2^	1.09	1.05–1.13	<0.001	1.03	0.98–1.08	0.249
Current smoking, yes or no	1.15	0.86–1.55	0.338	1.04	0.72–1.51	0.840
Alcohol drinking, yes or no	0.76	0.60–0.96	0.019	0.77	0.59–1.00	0.047
Regular exercise, yes or no	1.43	1.13–1.80	0.002	1.19	0.94–1.52	0.151
Systolic blood pressure, mmHg	1.02	1.01–1.02	<0.001	1.00	0.99–1.02	0.885
Diastolic blood pressure, mmHg	1.02	1.01–1.03	<0.001	0.99	0.97–1.02	0.501
Fasting plasma glucose, mg/dl	1.03	1.02–1.04	<0.001	1.01	1.00–1.02	0.048
C-reactive protein, mg/L	1.07	1.00–1.15	0.052	0.94	0.86–1.03	0.213
Hypertension medication, yes or no	3.22	2.44–4.24	<0.001	1.83	1.34–2.49	<0.001
TG/HDL-C quartiles, Q1 vs. Q4	2.99	2.08–4.29	<0.001	2.26	1.47–3.48	<0.001

**Table 4:** Hazard ratios and 95% confidence intervals for new-onset ischemic heart diseases according to TG/HDL-C quartiles.

	**TG/HDL-C quartiles**				***P*** **for trend**
	**Q1**	**Q2**	**Q3**	**Q4**	
New cases of ischemic heart disease, n	39	73	91	118	
Mean follow-up, years	2.4 ± 1.1	2.4 ± 1.1	2.4 ± 1.1	2.4 ± 1.1	
Pearson-years of follow-up	9,682	9,834	9,676	9,839	
Incidence rate/1,000 person –years	4.0	7.4	9.4	12.0	
Model 1	1.00 (reference)	1.64 (1.07–2.52)	1.85 (1.21–2.83)	2.35 (1.53–3.60)	0.001
Men	1.00 (reference)	1.41 (0.79–2.54)	1.55 (0.88–2.74)	1.89 (1.07–3.32)	0.137
Women	1.00 (reference)	1.85 (0.99–3.45)	2.14 (1.12–4.12)	2.98 (1.52–5.84)	0.017
Model 2	1.00 (reference)	1.63 (1.06–2.50)	1.82 (1.19–2.79)	2.30 (1.49–3.53)	0.002
Men	1.00 (reference)	1.41 (0.78–2.54)	1.55 (0.88–2.74)	1.86 (1.05–3.28)	0.164
Women	1.00 (reference)	1.85 (0.99–3.45)	2.10 (1.09–4.05)	2.95 (1.50–5.82)	0.020
Model 3	1.00 (reference)	1.63 (1.07–2.51)	1.82 (1.19–2.79)	2.26 (1.47–3.48)	0.003
Men	1.00 (reference)	1.42 (0.79–2.56)	1.54 (0.87–2.72)	1.81 (1.03–3.20)	0.209
Women	1.00 (reference)	1.86 (0.99–3.47)	2.13 (1.10–4.09)	2.98 (1.51–5.89)	0.019

*Model 1: adjusted for age, sex, body mass index, smoking status, alcohol intake, and physical activity*.

*Model 2: adjusted for age, sex, body mass index, smoking status, alcohol intake, physical activity, systolic blood pressure, diastolic blood pressure, fasting plasma glucose, and high sensitivity C-reactive protein*.

*Model 3: adjusted for age, sex, body mass index, smoking status, alcohol intake, physical activity, systolic blood pressure, diastolic blood pressure, fasting plasma glucose, high sensitivity C-reactive protein, and hypertension medication*.

### Pairwise Comparison With Metabolic Syndrome Parameters

Pairwise comparison of receiver operating characteristic (ROC) analysis for incident IHD revealed that the AUC of TG/HDL-C was significantly higher than that of metabolic syndrome (*P* < 0.001), which was similar to that of the number of metabolic syndrome components (*P* = 0.929). In the longitudinal model, the AUC, sensitivity, and specificity of TG/HDL-C were 0.607, 64.2, and 52.1%, respectively (Table 5).

**Table 5:** TG/HDL-C vs. metabolic syndrome and the number of metabolic syndrome components for predicting ischemic heart disease.

	**Pairwise comparison of AUC**			**Classifying ability for ischemic heart disease**			
	**Difference**	**95% CI**	***P*** **-value**	**Sensitivity (%)**	**Specificity (%)**	**AUC**	***P*** **-value**
TG/HDL-C vs. metabolic syndrome	0.059	0.031 to 0.087	<0.001				
TG/HDL-C vs. the number of metabolic syndrome components	0.001	−0.025 to 0.027	0.929				
The number of metabolic syndrome components vs. metabolic syndrome	0.064	0.037 to 0.083	<0.001				
TG/HDL-C				64.2	52.1	0.607	<0.001
Metabolic syndrome				21.5	88.1	0.548	<0.001
The number of metabolic syndrome components				79.1	37.4	0.608	<0.001

*AUC, area under the receiver operating characteristic curve*.

## Discussion

This large-scale cohort study on community-dwelling Koreans without diabetes over a 50-month period found that higher TG/HDL-C values were associated with IHD incidence, which remained after adjustment for lifestyle factors, inflammation, and hypertension medication. Moreover, compared with metabolic syndrome, TG/HDL-C had a more powerful predictive value for IHD.

The association between TG/HDL-C and incident IHD was more prominent in women than in men. Additionally, the HRs of incident IHD in women significantly increased according to the TG/HDL-C quartiles; contrastingly, those in men did not exhibit significant trends. Cheng et al. conducted a prospective study on 11,946 Chinese individuals without diabetes found that the risk for type 2 diabetes increased by approximately 30% with 1-unit of the TG/HDL-C increment (8). Moreover, they observed a clearer dose-response relationship in women than in men. Regarding cardiovascular disease (CVD), studies conducted in men and women separately have established this relationship. Hadaegh et al. (23) showed that Iranian men in the highest TG/HDL-C quartile had a 1.75-fold higher risk of coronary heart disease over a median follow-up period of 6.5 years compared with the reference low quartile. Another longitudinal study conducted by Bittner et al. reported a positive relationship between the TG/HDL-C and suspected CVD mortality in 554 US women over a median period of 6 years (16). The former and latter studies on men and women, respectively, reported a fourth quartile cut point of 6.9 and 2.8, respectively. Recent studies on TG/HDL-C have reported that it is associated with long-term mortality in high-risk individuals who have undergone percutaneous coronary angiography (24), as well as the relevance of subclinical coronary arterial calcification in low-risk individuals without diabetes (12). However, these studies did not address sex differences. Another study reported that TG/HDL-C, but not the LDL-C to HDL-C ratio, in women has a significant predictive value for CVD in a population-based cross-sectional study, unlike in men (25). Therefore, it may be advantageous for assessing and managing TG/HDL-C differently in men and women.

There was a noticeable increase and decrease in TG and HDL-C, respectively, with insulin resistance progression; moreover, the TG/HDL-c ratio is useful for assessing early insulin resistance (6, 26). High TG and low HDL-C are the two most common lipid abnormalities among Korean adults aged > 20 years, with a prevalence of approximately 28.7 and 41.2%, respectively (27). The Korea National Health and Nutrition Examination Survey indicated an increase in both high TG and low HDL-C by > 30% in 2010 compared with 2007. In the United States, ~30 and 40% of adult men /women in their 20's and 30's, respectively, have high TG and/or low HDL-C (28). Individuals with insulin resistance are more susceptible to atherosclerosis; moreover, coronary arterial disease can develop and progress from early insulin resistance (5, 29). The atherogenic link between TG/HDL-C and IHD, besides insulin resistance, is associated with the generation of small, dense LDL particles, which can cause vascular compromise (10, 30, 31). The NCEP guidelines for metabolic syndrome indicate well-established sex differences in the HDL-C level (32). In the same context, the TG/HDL-C ratio was significantly higher in men; however, its association with IHD was more pronounced in women.

A major strength of our study was that this was a longitudinal large-scale study linked to HIRA data, which are based on the universal coverage system in Korea. This decreases the chance of missing data. This study has several limitations. First, given that the study cohort comprised volunteers for health promotion screenings conducted at a single hospital, the participants could have been slightly healthier than the general Korean population. Moreover, some confounding variables, including dietary data and traditional insulin resistance markers, may have not been initially assessed. Second, some individuals with diabetes may have been included in the final analysis since glycated hemoglobin A1c assessment and oral glucose tolerance tests were not performed at the beginning of the study.

## Conclusions

An increased TG/HDL-C ratio precedes and significantly predicts future IHD among community-dwelling Koreans. Moreover, compared with metabolic syndrome, the TG/HDL-C was a more powerful predictive indicator of IHD. Additionally, there is a need to consider sex differences when interpreting TG/HDL-C, even among individuals without diabetes. A high TG/HDL-C ratio may be a useful indicator for assessing the CVD risk in adults at the preclinical stage.

## Data Availability Statement

The raw data supporting the conclusions of this article will be made available by the authors, without undue reservation.

## Ethics Statement

The studies involving human participants were reviewed and approved by The Institutional Review Board of Yonsei University College of Medicine. The patients/participants provided their written informed consent to participate in this study.

## Author Contributions

BP, DJ, and YL: study concept and design. BP, HL, and YL: acquisition, analysis and interpretation of data, and critical revision of the manuscript for important intellectual content. BP and DJ: drafting of the manuscript. All authors contributed to the article and approved the submitted version.

## Conflict of Interest

The authors declare that the research was conducted in the absence of any commercial or financial relationships that could be construed as a potential conflict of interest.

## Publisher's Note

All claims expressed in this article are solely those of the authors and do not necessarily represent those of their affiliated organizations, or those of the publisher, the editors and the reviewers. Any product that may be evaluated in this article, or claim that may be made by its manufacturer, is not guaranteed or endorsed by the publisher.
